# Role of Silver Nitrate Spray for Skin Wound Care in Patients with Toxic Epidermal Necrolysis: Our Experience in 4 Patients

**DOI:** 10.3390/life13122341

**Published:** 2023-12-14

**Authors:** Jose Dario Martinez, Jesus Alberto Cardenas, Manuel Soria, Luis Manuel Saenz, Kattya Estrada, Sergio Maximo Delgado, Marius-Anton Ionescu, Camelia Busila, Alin Laurentiu Tatu

**Affiliations:** 1 Department of Internal Medicine, Faculty of Medicine, University Hospital José Eleuterio González, University Autonomous of Nuevo León, Monterrey 66455, Mexico; 2 Department of Rheumatology, Faculty of Medicine, University Hospital José Eleuterio González, University Autonomous of Nuevo León, Monterrey 66455, Mexico; jesus.cardenasd@uanl.edu.mx; 3 Dermatology Department, Hospital Civil, Guadalajara 44630, Mexico; manuelspitz@hotmail.com; 4 Medical Students, Faculty of Medicine, University Hospital José Eleuterio González, University Autonomous of Nuevo León, Monterrey 64460, Mexico; luis.saenzmdn@uanl.edu.mx (L.M.S.); kattya.estradacns@uanl.edu.mx (K.E.); sergio.delgadoj@uanl.edu.mx (S.M.D.); 5 Department of Dermatology, University Hospital Saint Louis, 75010 Paris, France; dr.toni.ionescu@gmail.com; 6 “Sf. Ioan” Emergency Clinical Paediatric Hospital, 800179 Galati, Romania; 7 Clinical Medical Department, Faculty of Medicine and Pharmacy, “Dunarea de Jos” University, 800008 Galati, Romania; dralin_tatu@yahoo.com; 8 Dermatology Department, “SfantaCuvioasaParascheva” Hospital of Infectious Diseases, 800179 Galati, Romania; 9 Multidisciplinary Integrated Center of Dermatological Interface Research MIC-DIR (Centrul Integrat Multidisciplinar de Cercetare de InterfataDermatologica—CIM-CID), “Dunărea de Jos” University, 800201 Galati, Romania

**Keywords:** toxic epidermal necrolysis, Stevens–Johnson syndrome, silver nitrate, wound care

## Abstract

Stevens–Johnson syndrome (SJS) and toxic epidermal necrolysis (TEN) are examples of severe cutaneous adverse reactions to drugs (SCARs) with several international recommendations for global medical management, ranging from pharmacological systemic therapy to skin wound care. There is no defined best management of the skin wounds in SJS/TEN. The care of wounds is essential to initiate re-epithelialization. Our objective is to improve the cicatrization process, avoiding scarring due to deepening of the wounds, as well as prevent infections, achieve pain control, and avoid loss of serum proteins, fluids, and electrolytes. In this retrospective case series, we highlight the value of systemic therapy and the use of silver nitrate for wound management in four patients with TEN.

## 1. Introduction

SJS and TEN are drug-induced skin reactions categorized as severe cutaneous adverse reactions (SCARs), along with acute generalized exanthematous pustulosis (AGEP), drug reaction with eosinophilia and systemic symptoms (DRESS syndrome), and generalized bullous fixed drug eruptions (GBFDEs). SJS is differentiated from TEN based on the percentage of the body surface area (BSA) detachment, as shown in Table 1. Skin biopsy is essential to distinguish SJS/TEN from clinical mimics [1].

The incidence of SJS is 1.5–6.0 cases per million, with a prevalence across all age groups, affecting both genders (predominantly female), and is most commonly triggered by drugs with a latency period of 1–3 weeks. TEN is a life-threatening condition with an incidence of 0.4–1.9 cases per million and a high mortality rate (30–40%), especially in elderly patients with multiple comorbidities. Risk factors for SJS/TEN include HIV infection, mycoplasma infection, and advanced age. Genetic predisposition is evident in HLA B58:01 and HLA B15:02 haplotypes, associated with the use of allopurinol and carbamazepine, respectively [2].

## 2. Materials and Methods

A review was conducted from 2020 to 2023. The inclusion criteria consisted of a documented history of drug use associated with TEN, a confirmed clinical diagnosis, and detachment of more than 30% of BSA. Based on the patients’ comorbidities, we chose between intravenous dexamethasone, CyA and IVIG for systemic therapy, and for skin wound care, we used 0.5% silver nitrate solution sprayed over denuded areas twice a day.

## 3. Results

We included four patients diagnosed with TEN in our study (Table 2): three females and one male, aged between 3 and 57 years. We evaluated the drugs administered to these patients one month before the onset of symptoms using the Naranjo algorithm to determine the specific drug responsible for the adverse reaction. Three patients were admitted to a regular hospital room; one patient required intensive care unit (ICU) admission due to pulmonary affection that required supplementary oxygen and respiratory therapy. Both internal medicine and dermatology services participated in their treatment and management. Once patients were admitted, the culprit drug was suspended, SCORTEN (score for TEN severity) was calculated, and the patients’ comorbidities were assessed (as shown in Table 1). The shortest recorded hospital stay was 7 days, while the longest lasted 21 days, the latter occurring due to complications unrelated to skin wounds.

**Figure 1:**
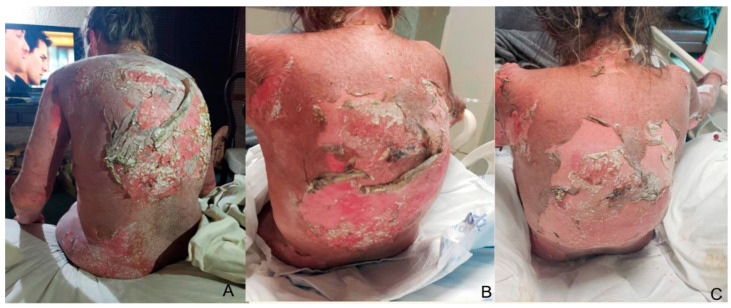
Extensive skin detachment before hospitalization (day 0) is noted in a 57-year-old female patient with TEN on her back (**A**). Treatment follow-up at the third day of BID silver nitrate application (**B**), and post-treatment photograph on day 7 before discharge with adequate re-epithelization of detached areas (**C**).

**Figure 2:**
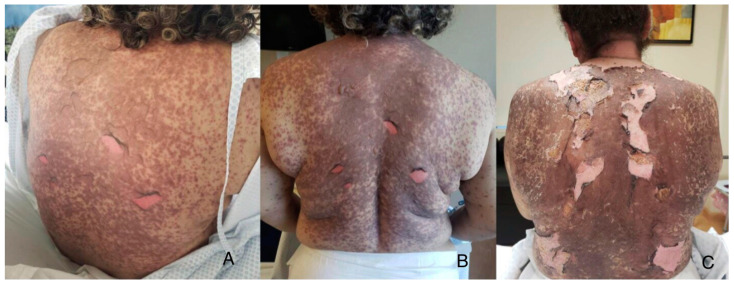
Treatment on the first day of hospitalization with extensive epidermal detachment on her back is noted in a 51-year-old female patient with TEN (**A**). Follow-up on day 5 showing dry skin after silver nitrate BID (**B**). Final photograph on day 15 before discharge with good clinical outcome (**C**).

We treated four patients with TEN, including three female patients: a 3-year-old treated with IV dexamethasone, two older patients (51 and 57 y/o) treated with IV dexamethasone + CyA, and a 28-year-old male treated with IV dexamethasone + IVIG. Our treatment approach involved identifying and discontinuing the offending drug, assessing for comorbidities, calculating SCORTEN, and evaluating the percentage of detached body surface area (BSA%). For wound care, we utilized silver nitrate in spray, a readily accessible and cheap treatment that minimizes direct contact with the wound and associated pain, whose use in dressings is reported in the scientific literature. We utilized silver nitrate twice daily until re-epithelialization was achieved, and on average, it took about 5–10 days (see Figure 1 and Figure 2). We did not use special dressings or blankets. We checked improvement in re-epithelialization by performing the Nikolsky sign test over the affected skin to avoid debridement. All four patients demonstrated a complete response to treatment and experienced only mild burning and no pain during the application. No significant adverse events related to silver absorption, toxicity, or local irritation were noted. Our patients had a fast response to the use of systemic therapy and silver nitrate together. Clinical characteristics, the offending drug, latent period, and other epidemiologic variables are depicted in Table 1. Figure 1 and Figure 2 illustrate the most representative cases.

## 4. Discussion

A recent systematic review and meta-analysis aimed at identifying antibiotics causing SJS/TEN revealed 38 studies involving 2917 patients. Sulfonamides caused 32% of the reactions, followed by penicillin in 22%, cephalosporins at 11%, fluoroquinolones at 4%, and macrolides at 2%. Approximately 28% of the reactions occurred with antibiotics, with sulfonamides being the primary culprits [3].

SJS/TEN are infrequent life-threatening drug eruptions with systemic symptoms and mucocutaneous involvement. While systemic symptoms are not uniformly present, they may precede skin and mucous membrane disease by 7 to 21 days [4]. These symptoms include pain (in the skin, eyes, lips, mouth, genital and anal), headache, rhinitis, malaise, sore throat, cough, and myalgias. TEN skin disease manifests as erythematous or violaceous patches, targetoid plaques, bullae, erosions, and necrosis. The bullae typically exhibit a positive Nikolsky sign and the Asboe-Hansen sign. Mucosal disease affects most SJS patients (present in >80% of cases), with oral sites more frequently affected than ocular, genital, or anal mucosa [1].

Disease severity and prognosis can be further delineated by using the SCORTEN criteria to assess the presence of risk factors that determine a score. A higher score means a higher mortality rate. SCORTEN should be calculated on day 1 and also on day 3 of hospitalization [1] (Table 3).

The diagnosis for SJS/TEN is established through a skin biopsy, revealing apoptotic keratinocytes, epidermal necrosis, basal vacuolization, and lymphohistiocytic dermal/perivascular infiltration.

Drug hypersensitivity can result in various types of reactions. In most cases, drug hypersensitivity presents as a generalized maculopapular rash, which is typically mild and self-limiting. The differential diagnosis of SJS/TEN should include other SCARs such as DRESS syndrome, AGEP, and GBFDE [5,6].

To manage a patient exhibiting signs of SJS/TEN, a multidisciplinary approach is essential. Dermatology, internal medicine, intensive care medicine, nutrition experts, ophthalmology, wound care specialists and other specialties should collaborate on a plan of action. Studies have shown that when patients are transferred to the intensive care unit or burn unit in a timely manner, there is a significant decrease in mortality.

The management of hospitalized patients includes supportive therapy, such as maintaining room temperature, fluid replacement/resuscitation, stress ulcer prophylaxis, and prophylactic anticoagulation. Medical management implies ensuring a patient’s airway, providing enteral or parenteral nutrition, wound care, eye care, oral cavity care, genital care, urinary catheterization, renal surveillance, and pain control, usually involving opioids [7].

There is no international consensus regarding the potential addition of pharmacologic therapy in addition to supportive care, only guidelines of several dermatological associations from around the world. The use of systemic corticosteroids, intravenous immunoglobulin (IVIG), and TNF-α inhibitors such as etanercept and adalimumab remains a topic of debate. A recent Delphi based consensus only recommends supportive therapy [8].

Systemic therapy with IV steroids and cyclosporine A (CyA) has been shown to increase survival in patients with SJS/TEN, whereas other therapies such as thalidomide, IVIG, plasmapheresis or cyclophosphamide did not demonstrate this effect [9].

When diagnosing SJS/TEN, patients should be admitted to an intensive care unit (ICU) or Burn Center to initiate first-line medical therapy. First-line pharmacological therapy in many centers is systemic steroids such as IV dexamethasone or IV methylprednisolone unless there is an obvious or suspected infection, in which case other systemic therapies should be considered.

CyA is a calcineurin inhibitor that decreases TCD8 lymphocyte levels in the epidermis and inhibits granulysin, Fas ligand, and anti-TNF-α function. CyA has been shown to accelerate re-epithelialization and be a safe and effective systemic therapy in a study involving 71 patients with SJS/TEN [10,11].

TNF-alpha therapy for SJS-TEN is based on elevated TNF levels in bullae fluid. A study demonstrated that etanercept reduces hospital stay and has a steroid-sparing effect, without any demonstrated adverse effects after 6 months of follow-up after treatment [12]. Another recent multicenter observational study published in 2022 comparing corticosteroid monotherapy vs. etanercept + IV steroids revealed that the combination was more effective and safer for the treatment of SJS/TEN [13].

In a retrospective review conducted in 2022, all patients diagnosed with TEN and treated at the same center between 2017 and 2021 were analyzed. The study compared the efficacy of adalimumab to other treatment options, including systemic steroids, CyA, and IVIG. The results showed that all patients who received adalimumab experienced successful resolution of their condition. Furthermore, these patients had a significant reduction in hospitalization duration, with an average stay of 22.5 days compared to 33 days for those treated with alternative therapies [14].

The use of IVIG remains controversial. A meta-analysis from 2017, which included nine studies comparing support therapy vs. IVIG, revealed no difference in mortality [9].

Currently, there are no definitive guidelines for wound management in patients with SJS/TEN. However, it is generally recommended to minimize frequent wound dressing changes and patient manipulation, as these can interfere with wound healing and re-epithelization. The use of fluidized-air beds is recommended to minimize damage to the epidermis, and the application of mild emollients, such as petroleum-based products, is recommended for the affected area. Antimicrobial topicals should only be applied to areas of epithelial detachment [15].

Patients receiving nanocrystalline silver dressings require fewer dressing changes because they penetrate the skin better and patients are less likely to develop sepsis compared to patients receiving silver nitrate solution dressing [16]. The ability of certain silver dressings to exhibit rapid bactericidal activity against antibiotic-resistant organisms is critical in an era of antimicrobial resistance. The efficiency of silver dressings for the destruction of antibiotic-resistant bacteria has been demonstrated in vitro [1].

In a retrospective study conducted in 2018, the objective was to explore the absorption of silver in patients with SJS/TEN who were treated with silver nylon and silver foam dressings. The analysis included 14 patients, and the findings revealed noteworthy results. Out of the 14 patients evaluated, 5 individuals exhibited elevated serum silver levels ranging from 9.9 to 11.0 μg/L, surpassing the normal range of <5 μg/L. It is important to note that all of these patients experienced a decline in their white blood cell counts in comparison to their baseline levels. Therefore, if acute leukopenia or elevated ASL/AST is observed, it is recommended to measure serum silver levels to rule out silver toxicity [17,18].

## 5. Conclusions

Severe cutaneous reactions to drugs, such as SJS, TEN, and overlap SJS/TEN, are characterized by extensive epidermal detachment due to epidermal necrosis. These reactions are commonly triggered by multiple drugs, including antibiotics, antiepileptics, NSAIDs, HIV drugs, and even contrast media [15]. The use of systemic corticosteroids, CyA, IVIG, and anti-TNF-α can be debatable. Wound care, pain control, nutritional support, and fluid/electrolyte replacement, among other supportive care measures, continue to be the mainstay of treatment. Wound care recommendations exhibit significant diversity in SJS/TEN guidelines, and no universal consensus currently exists [7,15]. Preservation of non-viable epidermis and anti-shear skin care has been increasingly recommended. However, the application of dressings and topical drugs remains challenging in these acute painful conditions, as it sometimes needs to be carried out under general anesthesia or deep sedation, which can be harmful to the patient. The advantages of using silver nitrate include its antibacterial activity and promotion of neovascularization, accelerating healing. However, its disadvantages include demonstrated in vitro cytotoxicity and the potential systemic absorption of silver when large areas of the skin are involved [15].

In addition to pharmacological therapy, proper care of the skin wounds is crucial for the positive outcome of these patients. We propose the use of 0.5% silver nitrate (off-label) as a valuable approach in managing TEN and SJS/TEN overlap cases. Skin wound care involves spraying 0.5% silver nitrate twice a day over denuded skin. None of our patients develop any systemic toxicity or adverse skin reactions. We did not measure silver nitrate levels but instead monitored potential changes in laboratory blood values. The use of sulfadiazine is not recommended due to its relationship with SJS/TEN. We refrained from using special blankets or dressings on our patients because they are costly, difficult to apply, and painful to remove. Instead, we utilized the non-viable epidermis as a biological dressing. Death skin debridement at the right moment may be beneficial in certain cases, although our patients did not require this procedure due to our wound management approach. American Burn Association (ABA) guidelines published in 2008 suggested the debridement of denuded epidermis but also presented an alternative option using the epidermis as a biological dressing, advocating a conservative approach to wound care [19]. A study conducted in France by the French National Reference Center on toxic bullous dermatoses does not recommend removing detached skin. In contrast, a 2017 retrospective study involving a total of 32 patients with TEN and 10 with SJS/TEN overlap revealed that intensive wound management which included debridement of blistered epidermis and closure of the wound with synthetic and biological dressings resulted in benefits in mortality [20,21]. There is no widely accepted guideline supporting surgical debridement of vesicles, bullae, and non-viable epidermis in these patients, but it can be debated. An alternative would be to aspirate the bulla and leave the non-viable epidermis as a biological dressing using antiseptic agents. Leaving non-viable skin in place creates a biological dressing, which has created an anti-shear therapeutic approach. The goal of wound management in these patients should be to minimize physical manipulation to avoid pain, reduce the frequency of dressing changes in this painful situation, and mitigate the risk of infection [15,22]. Following these recommendations, we managed our patients without debridement and utilized silver nitrate 0.5% solution on the skin wounds, using the non-viable epidermis as a biological dressing, which generated favorable results. Topical silver nitrate has been proved to be a safe and effective therapy in a multi-center study assessing its efficacy in burns [23]. Silver nitrate possesses cauterizing and wound healing properties, with a low risk of functional disorders and lasting tissue damage [24,25].

In conclusion, the management of the skin with silver nitrate solution, supportive therapy, and systemic therapy is an excellent combination to accelerate wound healing and re-epithelialization in these patients. Given the longstanding use of silver nitrate in dressings for these cases, we suggest that the use of silver nitrate 0.5% in spray directly to the skin is useful in the management of TEN and SJS/TEN overlap cases. Skin wound care includes silver nitrate 0.5% in distilled water, as it is cost-effective, promising, and, based on our experience, a safe and effective alternative for skin wound care. We also observed a faster response compared to silver nitrate dressings used in the past for other patients. However, further research is needed to assess and demonstrate its efficacy and safety. It is important to acknowledge the limitations of our study, including the small sample size.

## Figures and Tables

**Table 1:** Classification for SJS/TEN. BSA: body surface area.

Classification	Percent of Body Surface Area with Epidermal Detachment
SJS	<10%
SJS/TEN overlap	10–30%
TEN	>30%

**Table 2:** Clinical features in four patients with TEN.

Gender	Female	Female	Female	Male
Age	3 y/o	51 y/o	57 y/o	28 y/o
Latency	2 weeks	3.2 weeks	2.4 weeks	2.2 weeks
Culprit drug	Ibuprofen	Carbamazepine	Moxifloxacin	Carbamazepine
Systemic therapy	Dexamethasone	Dexamethasone + CyA	Dexamethasone + CyA	Dexamethasone + IVIG
HIV infection	-	-	-	-
Comorbidities	None	TN	COPD	Epilepsy
SCORTEN	2	3	3	3
Total BSA affected/detachment %	80/40	90/50	80/50	90/40
Prodromal period	+	+	+	+
Mucosal involvement	+	+	+	+
Genital involvement	+	+	+	+
Ocular involvement	+	+	+	+
Rash	+	+	+	+
Hospital stay	7 days	15 days	7 days	21 days
Laboratory findings	-	-	-	-

CyA: cyclosporine, IVIG: intravenous globulin, TN: trigeminal neuralgia, COPD: chronic obstructive pulmonary disease, SCORTEN: score of toxic epidermal necrolysis, TBSA: total body surface area.

**Table 3:** SCORTEN and TEN risk mortality.

Parameter	Value for SCORTEN (1 Point)
Age	>40 years
Cancer	Yes
Percentage of skin detachment	>10%
Pulse rate	>120/min
Serum bicarbonate	<20 meq/L
Blood urea nitrogen	>28 mg/dL
Glycemia	>252 mg/dL
**Total score**	**Estimated risk of death in the acute phase**
0–1	3%
2	12%
3	35%
4	58%
>5	90%

## Data Availability

The data included in the manuscript cannot be shared publicly due to the need to protect the privacy of the included subjects. Data may be shared upon reasonable request to the corresponding author.

## Abbreviations

SJS	Stevens–Johnson Syndrome
TEN	Toxic epidermal necrolysis
SCARs	Severe cutaneous adverse reactions
AGEP	Acute generalized exanthematous pustulosis
GBFDE	Generalized bullous fixed drug eruption
HIV	Human immunodeficiency virus
HLA	Human leukocyte antigens
BSA	Body surface area
NSAIDs	Non-steroidal anti-inflammatory drugs
